# Perceptions of doctors and nurses at a Ugandan hospital regarding the introduction and use of the South African Triage Scale

**DOI:** 10.4102/phcfm.v8i1.1056

**Published:** 2016-03-29

**Authors:** Francis Mulindwa, Julia Blitz

**Affiliations:** 1 Faculty of Medicine and Health Sciences, Division of Family Medicine and Primary Care, Stellenbosch University, South Africa

## Abstract

**Background:**

International Hospital Kampala (IHK) experienced a challenge with how to standardise the triaging and sorting of patients. There was no triage tool to help to prioritise which patients to attend to first, with very sick patient often being missed.

**Aim and setting:**

To explore whether the introduction of the South African Triage Scale (SATS) was seen as valuable and sustainable by the IHK’s outpatient department and emergency unit (OPD and EU) staff.

**Methods:**

The study used qualitative methods to introduce SATS in the OPD and EU at IHK and to obtain the perceptions of doctors and nurses who had used it for 3–6 months on its applicability and sustainability. Specific questions about challenges faced prior to its introduction, strengths and weaknesses of the triage tool, the impact it had on staff practices, and their recommendations on the continued use of the tool were asked. In-depth interviews were conducted with 4 doctors and 12 nurses.

**Results:**

SATS was found to be necessary, applicable and recommended for use in the IHK setting. It improved the sorting of patients, as well as nurse-patient and nurse-doctor communication. The IHK OPD & EU staff attained new skills, with nurses becoming more involved in-patient care. It is possibly also useful in telephone triaging and planning of hospital staffing.

**Conclusion:**

Adequate nurse staffing, a computer application for automated coding of patients, and regular training would encourage consistent use and sustainability of SATS. Setting up a hospital committee to review signs and symptoms would increase acceptability and sustainability. SATS is valuable in the IHK setting because it improved overall efficiency of triaging and care, with significantly more strengths than weaknesses.

## Introduction

The practice of triage dates back to the 18th century. In World War 1 especially, military surgeons developed and implemented triage rules to promptly evaluate and categorise wounded soldiers. Triaging, or the sorting of patients, aims to bringing the greatest benefit to the highest possible number of patients by prioritising them, so that a patient with the greatest need is helped first.^1^ Triage and emergency care has always been one of the weakest and least emphasised components of healthcare systems in Africa and yet, if organised, can lead to saving many lives and reducing the ultimate costs of care.^2^

Many developing countries have been grappling with ever-increasing emergency and trauma cases in a setting of limited and dwindling resources for emergency care.^3,4,5^ Ensuring that the emergency care systems of such countries is up to expectations and demands has not been helped by the absence of plausible means of measuring and assessing the effectiveness of their emergency care systems.^6,7^ Many hospitals in low-income countries, with Uganda and International Hospital Kampala (IHK) no exception, lack a formal triage system, which has led to dangerous yet avoidable delays in the management of critically ill patients.^8,9,10^

There are many triage tools in place around the world, such as the Manchester Triage System (MTS), Canadian Triage and Acuity Scale (CTAS), Australian Triage Scale (ATS), Kampala Trauma Score (KTS), VitalPAC Early Warning Score (ViEWS), and Modified Early Warning Score (MEWS). The latter two have been successfully used in studies in Uganda and Tanzania as in-hospital predictors of the risk of patients dying but not as triage tools in the outpatient department and emergency unit (OPD and EU).^11,12^ The KTS, focussed on trauma management, was found difficult to use for individual patient triage and unsuitable for use as a screening tool in Malawi.^13,14^ Hence there is a need for a triage tool to help with emergency care and treatment of very sick patients.^15,16^ The South African Triage Scale (SATS) has been found to be user friendly and more suitable for use in developing countries, following successful use in Southern Africa. Triaging with the SATS involves asking for presenting complaints, looking for clinical signs and measuring vital signs which are then used in calculating the triage early warning score (TEWS).^17^

The Ugandan healthcare system has the challenges of a dysfunctional referral system, poorly integrated services, attrition of public sector health workers, and inadequate financial and human resources.^18^ Uganda’s emergency care system has not been an exception to these challenges, with unco-ordinated and poor ambulance services and insufficient emergency room infrastructures. Frequently, patients find their way to a hospital’s emergency room, but very few hospitals have the emergency facilities to take care of their urgent condition, let alone a structured triage tool to sort them. If a basic component of healthcare, such as triaging, is not performed correctly, the other primary, secondary and tertiary aspects of care are hindered.

IHK, one of Uganda’s leading private healthcare providers, has a fully functional and fairly well-equipped emergency unit with a staff fully committed to providing improved services to the community. The hospital has for the past three years been using the MEWS for its in-patients. There has not been any tool used in the OPD and EU for triaging patients, who were being seen on a first-come first-serve basis and at the discretion of the health workers on whether they were seriously ill or not; hence the need for a practicable triage tool.

A survey on the perceptions of doctors and nurses regarding the implementation of SATS in South Africa found that 90% of the respondents rated the tool as acceptable.^19^ If SATS is to be used in Uganda, it is necessary to introduce and assess its applicability in the Ugandan setting. Introducing this validated tool successfully would not only open the way for its countrywide use but also provide an opportunity for future assessment of the effectiveness of the hospital’s emergency and primary healthcare units.^20,21,22,23^ The present study therefore set out to explore whether the introduction of SATS is seen as worthwhile and sustainable by the staff of the IHK OPD and EU. The IHK clinical managers appreciated the importance of using a standardised triage tool in the emergency unit and thus encouraged the introduction of SATS.

## Research methods and design

### Study design

The study was conducted using a qualitative research methodology.

The researcher initially introduced SATS to the Director of Clinical Services and Nurse In-charge of the IHK OPD and EU. It was agreed during these discussions that, to adopt the tool to the IHK triaging system, green, yellow, orange and red stickers should be used to represent the different codes. On triaging and coding a patient, a matching colour sticker would be put on the personal identifying information (PII) printout and the triage time recorded on the sticker. Over a week, two initial one-hour training sessions were held for nurses and doctors working in the OPD and EU, which enabled those working on different days and shifts to attend. Thereafter the tool was made available. A separate one-hour training session was also held for doctors working in other departments (e.g. internal medicine, women’s health) during one of the doctors’ continuing medical education (CME) meetings. Individual information sessions were held with the heads and members of the various hospital departments, such as reception, laboratory and in-patients, to raise awareness of the tool.

A clinical audit was done after five weeks’ use of SATS to improve on the consistency of its use, with structural, process and outcome criteria considered. The audit showed that the rate of consistent use of the tool by nurses in the EU was low, at about 10%. This was attributed to the minimal and inadequate support from the administrative staff and the implementation processes, with many of the structural components not in place. Also noted was the need for more training. The results were discussed with the OPD and EU nurses and doctors, and the recommended interventions implemented. A glucometer, SATS posters, manuals, triage register and whiteboard were then put in place. A follow-up one-hour training session was done to ensure consistency.

### Setting

IHK, one of the few fairly well-resourced Ugandan hospitals, is a secondary hospital whose OPD and EU take care of patients from all over the country. There are in total 10 medical officers and 25 nurses working in the IHK OPD and EU. The dayshift has 2–4 nurses in the EU and 1 or 2 nurses in the OPD. The nightshift has 2–3 nurses in OPD & EU. One doctor is allocated to the EU at any one time, with 3–4 during the OPD dayshift. Up to 40 patients are seen in the EU and 100 in OPD on a busy dayshift, and 10–40 respectively during a light shift. The OPD and EU nightshift can have between 5 and 20 patients. In the absence of a triage tool, the doctors and nurses use their own clinical judgment to determine the order for patients to be attended to. Very sick patients are usually seen in the EU whilst the more stable are seen in the OPD.

### Study population

The study population consisted of all fulltime-employed doctors and nurses working in the IHK OPD and EU with work experience of between 1 and 6 years.

### Sample size

The study population was 35 members of staff, comprising 10 doctors and 25 nurses. Data were collected by in-depth interviews of 12 nurses and 4 doctors working in the OPD and EU who were randomly selected by choosing every second person in an alphabetical list of surnames.

### Data collection

After 6 months of continuous use of SATS, data were collected by the researcher over 2 weeks, using the semi-structured in-depth interviews of the nurses and doctors. Respondents were given anonymous identifiers, namely D for doctors and N for nurses, with numerical labels. The interviews were audio-recorded on a tape recorder and handwritten notes also taken. The recordings were then transcribed.

### Data analysis

Data were analysed using content analysis. The data were checked, and made familiar by reading the observation notes and listening to the tapes. The recordings were transcribed, and mistakes were corrected. The data were then coded with the categories formed, aligned with aims and objectives, indexed, charted themes interpreted and confirmed, and the results presented. The researcher was aware of his own reactions when interpreting the data.^22^ Computer programme ATLAS.ti was used for coding and collating of data.

### Ethical considerations

Permission to conduct the study was obtained from the IHK Director of Clinical Services, with the study reviewed by the Health Research Ethics Committee (HREC) of Stellenbosch University (Ethics Reference Number: S14/03/071).

## Results

### Respondents’ findings

#### Challenges encountered before introduction of South African Triage Scale

Table 1 summarises the demographic profiles of the 16 participants in the study. Most respondents acknowledged that they were not able to appropriately prioritise which patients to attend to first without a suitable triage tool. Frequently, stable patients were seen first whilst very ill patients quietly deteriorated in the waiting queue. Many respondents mentioned that there was no strong scientific reasoning for health workers to use in explaining extended waiting times to patients, and why some patients were seen earlier than the rest. In the previous triaging system, the superficial interactions of health workers with patients led to the occasional misdiagnosis. There was no structured way to alert other departments, such as the laboratory, on how certain samples or patients needed to be urgently worked on. An EU nurse pointed out:

‘… before the tool was introduced, we’d just triage … we didn’t have anything like to rate how severe and un-severe the patient is.’ (N9)

**TABLE 1:** Demographics of respondents who participated in the study.

Designation	Sex		Shift		Unit	
		
	Femaleh	Male	Dayshift	Nightshift	OPD	EU
Nurse	11	1	11	1	2	10
Doctor	1	3	3	1	1	3

**Sub-total**	**12**	**4**	**14**	**2**	**3**	**13**

#### Identified strengths of South African Triage Scale

Respondents said that the SATS brings more clarity in the event of a medico-legal case or administrative issues following a patient filing a complaint. A number of respondents outlined that the SATS was so easy to use that even receptionists who had no medical knowledge could detect symptoms and direct patients to either the OPD or EU. There were respondents who reckoned that the SATS was comprehensive, including clinical symptoms, quantifiable vital signs scores and investigations, which is a strength.

A doctor thought the SATS can be helpful in triaging by phone during ambulance calls to determine whether an ambulance is sent or not. He said:

‘… this can also help us with ambulance calls, even on a phone call you can actually try to grade where a patient falls.’ (D1)

Most respondents said the SATS was applicable to the IHK and Ugandan setting, which is a big strength. One nurse and doctor thought that the provision to re-assess and rescore patients when not seen within the allotted time was a strength. There were respondents who thought that SATS was helpful in planning care and staffing levels in the EU and in-patients, depending on the severity of patients’ conditions. A nurse working in the EU said:

‘… Someone looking at the board [*in the EU or inpatients*] before even they go to see the patient physically would know we shall need maybe five nurses to attend to this ward or maybe an extra doctor …’ (N4)

#### Weaknesses and/or challenges faced during the use of South African Triage Scale

Nurses and doctors both highlighted the challenge of inconsistent use of the tool by triage nurses, which was attributed to either disinterest or OPD and EU coverage by nurses from other departments. More than half of the respondents thought the tool was time-consuming, especially with few staff at peak hours, as it required thoroughness with many steps involved such as history taking, TEWS calculations and critical analysis. Additionally, green-coded patients did not want to wait long and requested their codes to be changed to more urgent colours. At one time, the hospital delayed in acquiring stickers, which led to occasional stock-out, especially of green stickers. One respondent mentioned that the similarity between the red and orange colour stickers was sometimes confusing, and hence the need for vigilance, otherwise patients would receive the wrong interventions:

‘… the colouring [*of stickers*] between red and orange is almost the same. Unless very keen, someone may fail to distinguish the two colors.’ (N1)

‘… other people were not minding about what you mean by the [*triage*] code …’ (D4)

A few respondents thought that the TEWS score of zero for a systolic blood pressure (SBP) > 170 was not representative of the nature of urgency it required. They continued treating these patients as for emergency. Other respondents thought that low-cadre nurses such as nursing assistants/auxiliary nurses (nurses with minimal nursing training < 4 months or trained on the job) found some difficulty in using the SATS tool and did not understand and could not explain it:

‘… the low cadre nurses (auxiliary nurses) use it at times with difficulty.’ (N2)

## How doctors and/or nurses perceive South African Triage Scale and its impact on their practice

Most respondents were appreciative of how SATS improved their pace and level of response to emergencies, with shortened times of stay for very ill patients in the EU. This enabled patients to receive timely interventions and potentially reduced complications. Most respondents said the tool improved communication between health workers. The resulting better patient handover, co-ordination and continuity of care led to an overall improvement in-patient care. All respondents maintained that SATS improved communication and interaction between patients and health workers, with rapport created and explanations given of the steps, which reduced complaints that patients had had with the previous system. An EU nurse said:

‘… it has helped the nurses to interact more with patients, to dig out, to know other issues. So it creates a better nurse-patient relationship.’ (N4)

Some respondents reiterated that SATS always gave health workers a sense of responsibility and accountability. Nurses became more involved in-patient care and shared the collective responsibility of patient care with doctors. Many respondents noted that reduced waiting times for very ill patients who needed urgent care led to fewer complaints. Consequently, doctors felt that they were able to prioritie their patients more effectively whilst acquiring a new skills set. A doctor attested to this, saying:

‘Skill wise, it has brought and made me learn many things.’ (D4)

‘… waiting time has really improved and giving us less complaints from our clients.’ (N1)

## Recommendations of doctors and nurses on the use of South African Triage Scale

Respondents reiterated the need for continuous training on the use of SATS for nurses and doctors working in the OPD and EU, especially in the orientation of new staff. They also thought that other hospital staff such as laboratory personnel, receptionists, radiographers and in-patient staff should be trained on the use of SATS because they worked largely with OPD and EU staff. One respondent recommended that an awareness programme for patients on SATS be put in place. A number of respondents were appreciative of the tool and recommended that it be introduced in other Ugandan hospitals. One EU nurse said:

‘It’s a very, very good and useful tool if we get the right training [*on*] how to use it.’ (N5)

Respondents suggested the creation of a computer application to automate patient coding as the hospital had an electronic medical records system which would make triaging easy. One EU nurse said:

‘… things that are automatic … where you enter those vitals in the computer, the TEWS should be just coming automatically.’ (N11)

A nurse respondent recommended an OPD and EU-designated ECG machine instead of sharing one with the cardiology unit. An OPD doctor mentioned that some tests such as glucometer and dipstick should be in the OPD triage station in addition to being in the EU. Another recommendation was for adequate staffing to ensure consistent use of the tool.

Some respondents recommended that a committee be set up to discuss the logic around the system of scoring and classification of signs and symptoms, with possible review if necessary. A nurse respondent said:

‘… some of the signs we take as emergency yet according to the SATS they are urgent, e.g. bleeding, fracture of the arm, difficulty in breathing. Can they be changed?’ (N6)

## Do staff see South African Triage Scale as worthwhile and sustainable?

Staff said that the tool, which is a requirement for the Council for Health Service Accreditation of Southern Africa (COHSASA accreditation that the hospital is undergoing, is good and not only needed in IHK but also other health facilities in Uganda. An OPD doctor said:

‘… it is very good, we need it here and I would love for this system to continue …’ (D1)

## Discussion

SATS was successfully introduced and evaluated in the present study and found to apply well to the IHK setting. Its introduction coincided with the hospital being in the process of acquiring COHSASA accreditation which requires a standard triage tool to be used in the EU. The respondents acknowledged that their skill sets had improved. They felt that the tool was a necessity and recommended its continued use and its introduction to other health facilities in Uganda.

The challenges previously experienced by the respondents whilst triaging patients were (1) inappropriate triaging of patients, which was associated with many patient complaints, (2) poor communication with and between health workers and patients, (3) missed diagnoses, and (4) delays in patients receiving much-needed care. All these challenges were addressed by the SATS introduction.

SATS was found to have considerably more strengths than weaknesses (Box 1). It is comprehensive yet easy to use, with the provision to re-triage if a patient had waited longer than the target time. Patients who needed urgent care were easily identified, their waiting times reduced, diagnoses made and the chances of complications reduced. The tool also fostered good health worker-patient communication and that between health workers themselves. Nurses took up more responsibilities which had been previously taken to be that of doctors. The tool was useful in administrative issues stemming from patients’ complaints regarding their care. It was useful in guiding other hospital departments on the level of care required by patients from the EU and planning staffing, depending on the category of patients admitted. SATS could be used in telephonic triaging during ambulance calls. The efficiency of the respondents’ response to emergencies was improved by the tool.

**BOX 1:** SWOT analysis of findings from the interviews regarding possible implementation of South African Triage Scale in International Hospital Kampala.

**Factors Internal**	**Strengths** Easy to use and applicable to the IHK/Ugandan setting. Comprehensive with quantifiable scores complemented with physical signs and investigations. Ability to re-assess and rescore patients. Reduces waiting times of very sick patients. Enhances better diagnosis making and healthworkers’ patient care skills. Enhances good health worker-patient communication and between health workers themselves. Encourages nurses’ involvement in-patient care; hence sense of responsibility and accountability. Increases efficiency and pace of response to emergencies; hence reduces complications. Useful in resolving medico-legal/administrative concerns, workload staffing planning and possibly telephonic triaging. Guides other hospital departments in providing timely services to patients.	**Weaknesses** Time-consuming. Confusingly similar red and orange colour codes. Low TEWS score for SBP 170 which should be given a higher score. Controlled haemorrhage, closed fracture and breathing difficulty coded yellow instead of orange or red. Difficult to use by low-cadre nurses such as auxiliary nurses, nursing assistants.
**External**	**Opportunities** Need for the tool to be adopted in IHK and other health facilities in Uganda. Is one of the requirements for COHSASA accreditation that the hospital is in the process of acquiring, and is hence sustainable. Regular training of all IHK staff. Adjustment of the BP discrepancy of 170 scoring zero. Create a computer application that would automatically score and code patients. A team should be formed to re-evaluate signs and symptoms such as SBP, controlled haemorrhage, and closed fracture, and add more symptoms. Designated ECG machine in the EU and tests in the triage station. Adequate nurse staffing to allow consistent use of SATS. Provide educational and reading material for patients on SATS. Streamline the procurement processes for supplies required for using SATS.	**Threats** Inconsistent use of tools by nurses/doctors. Inconsistent supply of colour stickers by the hospital. Stable green-coded patients not prepared to wait. Low nurse staffing. Low patient awareness of the tool. SATS, South African Triage Scale; IHK, International Hospital Kampala; EU, emergency unit; COHSASA, Council for Health Service Accreditation of Southern Africa.

SATS, South African Triage Scale; IHK, International Hospital Kampala; EU, emergency unit; COHSASA, Council for Health Service Accreditation of Southern Africa.

MacLeod et al.^13^ and Haac et al.^14^ found the use of KTS as a triage tool to be limited, whereas the present study found SATS to be comprehensive and applicable to the IHK setting.

The present study agreed with Augustyn et al.^19^ on improved patient sorting following implementation of SATS. Whilst Augustyn et al.^19^ recommended that only nurses should prioritise patients, as is the case in IHK, the study found that receptionists still had a role in triaging as they were in most cases the first to come into contact with patients.

Whilst the SATS manual outlined that the triage provider can be a medical officer, registered nurse, enrolled nurse or enrolled nurse assistant, a few respondents in the present study thought that nurse assistants and auxiliary nurses used the tool with some difficulty. This study is in agreement with Augustyn et al.^25,26^ who said that the person with more training or experience must be in charge of triaging, which would in this case be a senior nurse or doctor overseeing the nursing assistants’ and auxiliary nurses’ activities. Similarly to Augustyn et al.^25,26^ the present study re-iterates the issue of having adequate nursing staff for consistent use of SATS. Augustyn et al.^25,26^ said that interpersonal skills are required to enable nurses to communicate with patients and their relatives. Our study adds to the finding that SATS improves communication between health workers, their patients and other hospital staff.

Edwards et al.^27^ argued that triage requiring patients coded green to wait was a delay in them receiving care, and this was seen in the present study when non-urgent patients did not want to wait, and requested that their codes be changed to urgent colours.

A limitation to the present study was that SATS was used for only 6 months with 4 training sessions held. A clinical audit was done after 5 weeks of using the tool but a follow-up clinical audit was not done. There was a 2-week period when the OPD had run out of green colour code stickers because the hospital had delayed in procuring them.

There is little doubt that SATS is applicable to IHK, with the hospital having continued to use it to date, which is a reflection of its value. The recently attained COHSASA accreditation by IHK will ensure that the hospital consistently supplies color code stickers, posters and patient education materials to maintain the required standards.

## Conclusion and recommendations

The SATS triage tool is most useful, compared with the previous triage system used. An overall positive impact such as improved emergency response and communication was realised on the practices of IHK OPD and EU doctors and nurses in the period of its use. It is applicable to the IHK setting and has proven to be sustainable with continued use. Additional uses to triaging of the tool were identified by OPD and EU nurses and doctors. Its strengths by far outweighed its weaknesses, which gives a positive reflection of SATS being worthwhile and sustainable in IHK. The recommendations of nurses and doctors clearly support the continued use of the tool.

It is recommended that SATS use will be made sustainable by (1) more training provided to staff, especially the new ones; (2) special training for low-cadre nurses to ensure they are ‘up to speed’ with the rest; and (3) in-patient departments, laboratory, radiology and receptionists would also benefit from SATS orientation. In the future, creating an automated patient coding application and ensuring adequate nurse staffing would ease triaging and ensure consistent use of SATS.

There may be a need in the study setting to create a committee to review the possibilities of adding more symptoms and enhancing the urgency labeling of some parameters already in SATS. As IHK was in the frontline of the recent Ebola epidemic in Uganda, there might be a need to include symptoms of Ebola in SATS, which would ultimately protect both patients and staff, enhancing the already noted worth of the tool.

A study estimating the nurse-hours spent on every category of patient such as red, orange, yellow and green, would help in planning ward or unit staffing. Studies should be done on a computer application for automating the triaging process and use of SATS in telephonic triaging.

The Ugandan Ministry of Health should consider adopting this tool, after testing its viability in a low-resource hospital setting. This could improve the emergency care of patients in all hospitals in the country. It would also be important to revise the triaging component in the training curriculum of Ugandan health workers.

## Appendix 1

### In-depth interview questionnaire

1. Having used the South African Triage Scale (SATS) as a triage tool in the IHK-EU, how did you find the use of this tool?
2. What did you think were the strengths of the SATS triage tool?
3. What were the weaknesses of the tool, and what challenges did you meet whilst using this tool?
4. What challenges did you identify in the IHK-EU/OPD triaging system before the introduction of this tool?
5. How has the use of the SATS triage tool affected your practice in the EU as a nurse/doctor?
6. What are your recommendations regarding the ongoing use of SATS in the IHK-EU and OPD?
7. What difference has SATS made to patient care?
8. What difference has SATS made to patient complaints?
